# Targeting the Roots of Recurrence: New Strategies for Eliminating Therapy-Resistant Breast Cancer Stem Cells

**DOI:** 10.3390/cancers13010054

**Published:** 2020-12-28

**Authors:** Margaret L. Dahn, Paola Marcato

**Affiliations:** 1 Department of Pathology, Dalhousie University, Halifax, NS B3H 4R2, Canada; meg.thomas@dal.ca; 2 Department of Microbiology and Immunology, Dalhousie University, Halifax, NS B3H 4R2, Canada

Cancer stem cells (CSCs) are functionally defined in our laboratories by their impressive tumor-generating and self-renewal capacity; clinically, CSCs are of interest because of their enhanced capacity to evade conventional therapies. Increased tumorigenicity and therapy resistance is a potent combination and strongly suggests that CSCs are involved in—if not responsible for—disease relapse. This Special Issue, “Breast Cancer Stem Cells: Therapy Resistance and Novel Therapeutic Targets” features a series of articles that contextualize the current literature on breast cancer stem cells (BCSCs), provide timely insights into how therapy resistance is cultivated by BCSCs and suggest how we may overcome that resistance.

Therapeutic resistance is a complex problem as it is not usually isolated to one specific subclass of drug but tends to include multiple drug classes. Multidrug resistance is a major hindrance to improving patient survival in all cancers. Perhaps an even greater concern, which current clinical strategies are only beginning to consider, is intratumoural heterogeneity and the crucial role it plays in dictating therapy resistance and recurrence [1]. Clonal evolution during the course of disease progression and treatment is only partially understood [2]. What is clear is that cells with stem-like characteristics are either selected-for or (troublingly) amplified by most conventional cancer therapies. 

In the classic Darwinian selection model, whereby only cells with intrinsic resistance can persist over the course of treatment, BCSCs are the survivors left to re-populate the tumor niche. Mechanisms that intrinsically aid BCSCs in surviving therapy include the well-characterized drug exporter proteins. Similar to normal stem cells, CSCs have enhanced efflux mechanisms, which in many cases is due to increased expression of ATP-binding cassette (ABC) transporters [3,4,5,6,7,8,9]. These transporters are also known to efflux chemotherapeutic drugs and are a common cause of chemotherapy resistance in breast cancer [10]. Another characteristic of BCSCs is endogenously high activity of aldehyde dehydrogenase (ALDH). Transformed breast cells have high ALDH1A1 levels [11]. Breast tumour samples with high levels of ALDH1A1 are associated with patient resistance to paclitaxel and epirubicin [12]. Patients with locally advanced breast cancer were treated with docetaxel and a combination of fluorouracil, epirubicin and cyclophosphamide (FEC 100); of the patients who did not have a complete response, if the remaining tumour cells were ALDH1A1 positive, this was strongly predictive of worse overall survival [13]. It is unclear whether these drugs are metabolized directly by ALDH enzymes or if ALDHs minimize their cellular toxicity by clearing reactive aldehydes generated during their primary mode of action. Alternatively, it is also possible that ALDH activity confers resistance by influencing cell signaling cascade such as the embryonic cell signaling pathways Notch and Hedgehog [14]. Embryonic signaling pathways such as wingless-related (Wnt), Notch, and Hedgehog have been implicated in therapeutic resistance for numerous cancer types [15,16,17,18].

In this Special Issue, the current state of research into therapeutic resistance in BCSCs is addressed. An underlying issue addressed by many of the articles is how to tackle breast cancer stem cell therapeutic resistance in the context of different clinical and molecular classifications of breast cancer. For instance, there is an undeniable focus on BCSCs within triple-negative breast cancer (TNBC), a subtype that is enriched for CSCs [19,20,21,22,23,24,25]. The overall survival for patients with metastatic TNBC has not improved in decades, and the standard therapy is still sequential single-agent chemotherapy [26]. This population desperately needs advances in therapeutic options, with many new strategies focused on BCSCs. Park et al. provide a comprehensive summary of similarities between a prototypical triple-negative breast cancer cell and BCSCs, including strikingly similar phenotypes in the functional assessments of “stemness” (namely the limiting dilution assay, mammosphere formation, and anoikis resistance) [27]. They propose that strategies for targeting BCSCs must be tailored to the clinical subtype, and that eliminating resident BCSCs in TNBC could lead to improved survival. Lee et al. take this stratification concept a step further by implying that molecular sub-groups within TNBC may also have distinct BCSCs with (distinct) therapeutic responses [28]. Indeed, stratification of TNBC into the luminal-androgen receptor (LAR), mesenchymal (MES), basal-like immunosuppressed (BLIS), and basal-like immune-activated (BLIA) sub-groups results in distinct drug sensitivities and suggests novel targeted therapeutic strategies. This was shown by Hill et al. wherein they demonstrated the value of targeting αvβ3 integrin in the mesenchymal sub-group of TNBC using novel peptide ψRGDechi [29]. A downside of the TNBC-focused research is that most studies have examined BCSCs in the context of chemotherapy or immunotherapy. These models provide few answers to those patients with hormone-receptor positive disease who develop resistance and recurrence to their hormone-based therapies. Rodriguez et al. review how BCSCs play a pivotal role in the acquisition of resistance to endocrine therapy in estrogen receptor positive (ER+) tumors [30]. Indeed, patients with ER+ tumors are at risk of late relapse up to twenty years from primary tumor removal, implying that there is a population of tumorigenic yet dormant cells that escape endocrine therapy and immune surveillance [31]. 

This issue of dormancy with subsequent re-activation of highly tumorigenic cells is the subject of De Angelis et al. with the viability of dormancy-targeted strategies for breast cancer dissected and challenged [31]. As with most BCSC-targeting strategies that modulate signaling pathways, targeting cellular dormancy may unleash the plasticity of BCSCs and transform them into highly aggressive and therapy-resistant metastatic cells. This underlines another core issue in the study of BCSCs: the plasticity of BCSCs makes them very challenging to study and even more challenging to treat [32]. This plasticity is perhaps most evidenced by the ease in which BCSCs move between epithelial and mesenchymal states during epithelial–mesenchymal transition (EMT), which is covered by several articles in this Special Issue [33]. Efficient EMT (and reverse MET) allows BCSCs to escape therapy, and such dynamic remodeling of the cellular phenotype is under the control of epigenetic mechanisms; or perhaps more controversially, cell fusion events. In this Special Issue, Batham et al. examine the role of the epigenetic modulator SET domain bifurcated histone lysine methyltransferase 1 (SETDB-1) in breast cancer metastasis [34]. Since drug resistance is transient, develops rapidly, and has diverse mechanisms, such epigenetic modifications are likely an essential component of BCSC drug resistance. Another feature that adds to cellular plasticity is an underappreciated phenomenon of tumor biology where cells fuse together to form hybrid cells. This is addressed by Hass et al. who discuss how heterofusions of mesenchymal stromal/stem-like cells with breast cancer cells contribute to the diversity of cell types present within a single tumor [35]. 

This Special Issue also proposes that we may need to re-visit our Darwinian selection model of cancer stem cell therapy resistance. Rodriguez et al. [30] suggest a more dynamic model of CSCs and discuss that in the context of endocrine therapy, treatment can induce the dedifferentiation of bulk cancer cells into BCSCs [30]. They suppose that CSC potential is a property of cancer, and that it is not clinically translatable—or perhaps even theoretically accurate—to depict CSCs as a distinct separate population of tumor cells. However, as discussed earlier, more research into ER+ BCSCs is warranted as most studies have been performed using the same MCF7 cell line.

The cellular heterogeneity of tumors and the microenvironment have been shown to be vital for BCSC maintenance and therapy resistance, and illustrate how important it is for future research to properly contextualize BCSCs in their environment [36]. Though mostly studied in the negative context of how cellular (e.g., cancer-associated fibroblasts, adipocytes, endothelial cells, and immune cells) and non-cellular (e.g., extracellular matrix, growth factors, and cytokines) elements of the tumor environment contribute to promoting stem-like cells and therapy resistance, the local tumor environment may actually enhance therapy in some contexts. In Melzer et al., they propose using mesenchymal stromal/stem-like cells as effective paclitaxel exosome factories to target breast cancer cells [37]. Paclitaxel uptake and release has also been observed in adipose stem cells and proposed as a drug delivery method [38].

In conclusion, as articulated in this Special Issue, creative solutions, such as harnessing the local cells as a drug delivery strategy, are required if we are to scale the technical and theoretical mountain of eliminating BCSCs. Most clinical trials with drugs targeting CSCs are summarized in this Special Issue, but as many in the field suggest, combinatorial and precision-medicine based strategies must be developed [27,28,32,38]. Scioli et al. review many such approaches and discuss treatment types that have not yet made it into the clinical realm such as gene targeted therapies, namely microRNAs [38]. Of interest, is a newer class of non-coding RNA: long non-coding RNAs (lncRNAs), which have functional relevance in BCSCs and have been proposed as novel BCSC-associated targets [39,40]. Undoubtedly, more research into BCSC therapy resistance is required, which will inform novel and effective therapies, with reduced recurrence.
